# Multidrug-Resistant Genotypes of *Plasmodium falciparum*, Myanmar

**DOI:** 10.3201/eid1703.100870

**Published:** 2011-03

**Authors:** Zhaoqing Yang, Chaoqun Li, Miao Miao, Zaixing Zhang, Xiaodong Sun, Hao Meng, Jie Li, Qi Fan, Liwang Cui

**Affiliations:** Author affiliations: Kunming Medical University, Yunnan, People’s Republic of China (Z. Yang, C. Li, J. Li);; Yunnan Institute of Parasitic Diseases, Pu’er, Yunnan, (Z. Zhang, X. Sun);; The Pennsylvania State University, University Park, Pennsylvania, USA (M. Miao, H. Meng, L. Cui);; Dalian Institute of Biotechnology, Dalian, Liaoning, People’s Republic of China (Q. Fan)

**Keywords:** Plasmodium falciparum, drug resistance, malaria, chloroquine, parasites, quinine, antifolate drugs, mefloquine, Myanmar, dispatch

## Abstract

We performed a molecular epidemiologic survey of mutations associated with drug-resistance genes in *Plasmodium falciparum* in northeastern Myanmar. In this region, 3 highly mutated drug-resistance haplotypes and 1 associated with decreased quinine susceptibility were prevalent, which suggests that parasites may be resistant to multiple commonly used antimalarial drugs.

Malaria is a major impediment to socioeconomic development in the Greater Mekong Subregion (GMS) of Southeast Asia (*1*). Malaria distribution in the GMS is extremely uneven, with areas of high endemicity in some countries and along international borders. In Myanmar, malaria is particularly problematic; more than half of malaria cases and approximately three fourths of malaria-related deaths in the GMS during 2007 occurred in Myanmar. The GMS has been the breeding ground of multidrug-resistant *Plasmodium falciparum*, and resistance to chloroquine and antifolates arose there and spread to Africa (*2**,**3*). In particular, recent detection of reduced artemisinin susceptibility at the Thailand–Cambodia border is a major concern (*4*). As a result, drug resistance has been monitored extensively in this region. In contrast, information about resistance to antimalarial drugs in Myanmar is exceptionally scarce. Accordingly, as our initial step toward a comprehensive antimalarial drug study in Myanmar, we performed a molecular survey of drug resistance in the northeastern region of this country.

## The Study

We screened by microscopy 4,980 patients with febrile illness who sought care at a malaria clinic in Kachin State, northeastern Myanmar, during 2007–2009; a total of 27.9% had malaria infections. *P. falciparum, P. vivax,* and mixed species infections accounted for 56.7%, 41.1%, and 2.2% of malaria cases, respectively. Finger-prick blood samples were obtained from 260 patients with uncomplicated *P. falciparum* infection who had not used antimalarial drugs during the previous 2 weeks. Parasite DNA was extracted from filter papers and genotyped at 3 polymorphic genes, which detected 54.6% of samples containing mixed-strain infections (*5*). Of the 118 samples with monoclonal infection, 117 samples were successfully genotyped by PCR and sequencing at 5 known and putative drug-resistance genes (Technical Appendix), some of which have been widely used for resistance surveillance and as predictors of clinical efficacy of antimalarial drugs.

**Figure 1:**
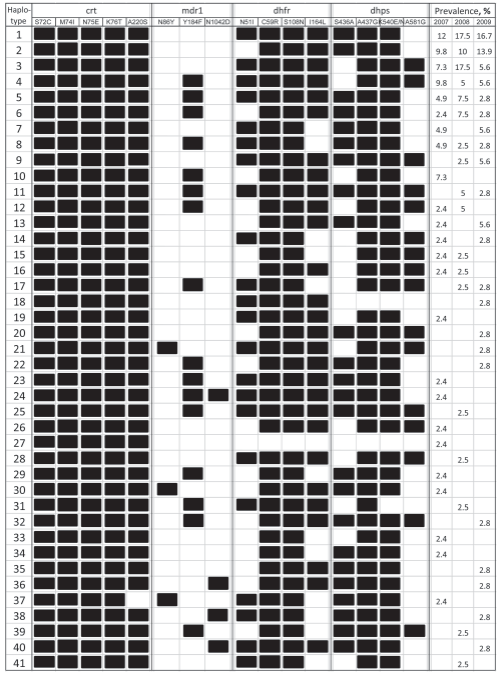
Multilocus genotypes in *Plasmodium falciparum* isolates, Kachin State, northeastern Myanmar, 2007–2009. A total of 41 haplotypes were identified from 117 parasite isolates. Wild-type and mutated amino acids are shown in white and black, respectively. Prevalence (%) of each multilocus genotype in each year is indicated in the right columns.

Sequencing of 2 fragments in the *P. falciparum* chloroquine resistance transporter (*pfcrt*) gene covering single nucleotide polymorphisms (SNPs) at codons 72–76 and 220, respectively (*6*), showed that the major chloroquine resistance determinant K76T mutation has reached fixation in the parasite population (Figure 1). All parasites had sequence CVIET at positions 72–76, compared with the wild-type sequence SVMNK. In addition, the A220S mutation associated with chloroquine resistance was predominant (99.1%).

**Figure 2:**
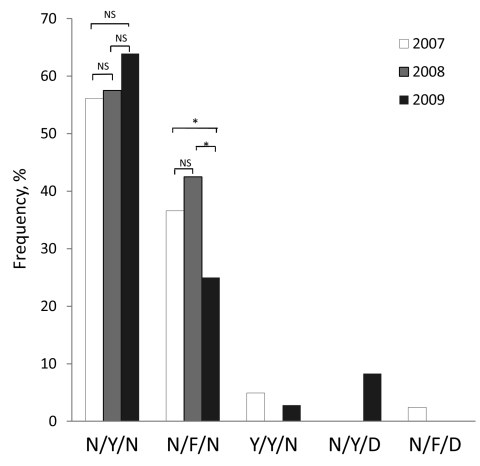
Annual prevalence of *Plasmodium falciparum* dihydrofolate reductase haplotypes among clinical samples collected from Kachin State, northeast Myanmar, 2007–2009. The x-axis shows the 5 haplotypes (the amino acids at positions 86, 184, and 1042 with mutated amino acids in **boldface**). The χ^2^ test was performed to compare prevalence of 2 major haplotypes between years. For each haplotype, NS denotes no significant difference (p>0.05) between years; asterisk (*) denotes significant difference (p<0.05) between years.

Sequencing of 2 *P. falciparum* multidrug resistance 1 (*pfmdr1*) fragments as described (*5*) detected only the N86Y, Y184F, and N1042D mutations making up 5 haplotypes (Figure 2). The overall haplotype prevalence differed significantly among the years (p<0.001, χ^2^ = 31.39, df = 8).The prevalence of wild-type haplotype was 59.0%. Among the 3 mutant codons, only Y184F reached a relatively high frequency (35.9%). One sample contained the double mutations 184F/1042D. Analysis of *pfmdr1* copy number from monoclonal infections by real-time PCR with 3D7 and Dd2 strains as negative and positive controls, respectively (*6*), detected no *pfmdr1* amplification.

Sequencing of the dihydrofolate reductase (*pfdhfr*) and dihydropoteroate synthase (*pfdhps*) genes (Technical Appendix) detected 4 SNPs in *pfdhfr* associated with pyrimethamine resistance (N51I, C59R, S108N, and I164L) and 4 SNPs in *pfdhps* associated with sulfadoxine resistance (S436A, A437G, K540E/N, and A581G) (Table 1). The overall haplotype prevalence of the 2 genes differed significantly between the years (p<0.0001, χ^2^ = 76.49, df = 28). Of the 5 *pfdhfr* haplotypes, wild-type NCSI was observed only in 1 sample in 2007; the remaining samples contained at least double mutations 59R/108N. Two triple-mutation haplotypes (N**RNL** and **IRN**I, mutations in **boldface**) were detected with N**RNL** being more frequent than **IRN**I in each year. Overall, quadruple mutations (**IRNL**) were found in >50% of the samples. In addition, frequency of triple and quadruple mutations increased gradually from 2007 to 2009. We found all 5 haplotypes in 2007 but only triple and quadruple mutations in 2009. In *pfdhps*, 10 haplotypes were found, and 437G and 540E/N mutations were highly prevalent: 98.3 and 96.6%, respectively (Table 1). Similarly, the wild-type *pfdhps* haplotype SGKA was found in only 2 samples. **AGE**A was the most common haplotype in each year and reached an overall frequency of 48.7%. Quadruple mutations (**AGEG)** were found only in 2008 and 2009.

**Table 1:** Prevalence of point mutation haplotypes in *pfdhfr* and *pfdhps* in clinical samples from Kachin State, northeast Myanmar, 2007–2009*

Gene	Haplotype	Codon†	Haplotype prevalence,‡ %		
			2007, n = 41	2008, n = 40	2009, n = 36
*Pfdhfr* (51, 59, 108, 164)	Wild-type	NCSI	2.4	–	–
	Double mutations	N**RN**I	9.8	5.0	–
	Triple mutations	N**RNL**	31.7^a^	25.0^b^	36.1^a^
		**IRN**I	14.6^a^	7.5^b^	16.7^a^
	Quadruple mutations	**IRNL**	41.5^a^	62.5^b^	47.2^a^
*Pfdhps* (436, 437, 540, 581)	Wild-type	SAKA	2.4	–	2.8
	Single mutation	S**G**KA	–	2.5	2.8
	Double mutations	S**GE**A	9.8	2.5	–
		S**GN**A	2.4	–	–
	Triple mutations	S**GEG**	26.8^a^	35.0^b^	19.4^c^
		S**GNG**	2.4	2.5	–
		**AGE**A	48.8^a^	45.0^a^	52.8^a^
		**AGN**A	7.3	–	8.3
	Quadruple mutations	**AGEG**	–	10.0	11.1
		**AGNG**	–	2.5	2.8

*Pfdhfr, *Plasmodium falciparum* dihydrofolate reductase; *Pfdhps,* *P. falciparum* dihydropoteroate synthase; –, no such haplotype detected. †Codons in **boldface** indicate mutated amino acids. ‡χ^2^ test was performed with all data points of the 2 genes, which showed that overall haplotype prevalence differed significantly between the years (χ^2^ test, p<0.0001, χ^2^ = 76.49, df = 28). Major *dhfr* and *dhps* haplotypes were individually compared. For each haplotype (in the same row), the labeling by different letters denotes significant difference between the years (χ^2^ test, p<0.05).

Molecular analysis of drug-resistance markers in monoclonal infections enabled us to obtain multilocus genotypes of the parasites. Genotyping each of the 117 parasite isolates at 16 drug resistance–related codons in the *pfcrt*, *pfmdr1*, *pfdhfr*, and *pfdhps* genes showed 41 haplotypes (Figure 1). Among these haplotypes, parasites containing >10 mutated codons accounted for 93.2% of the samples.

Polymorphisms in the minisatellite ms4760 of *P. falciparum* Na^+^/H^+^ exchanger (*pfnhe1*) are associated with quinine sensitivity (*5**,**7**–**10*). Sequencing of the *pfnhe1* fragment containing the ms4760 minisatellite from 79 monoclonal infections showed 10 haplotypes, with haplotype 7 the most predominant (54.4%) (Table 2). More than 64% of samples tested contained >3 copies of the DNNND repeat (Rep1); 76% contained 1 copy of the NHNDNHNNDDD repeat (Rep2). Accordingly, >60% of parasite isolates had a Rep1:Rep2 ratio of >3:1.

**Table 2:** Prevalence of the *pfnhe1* minisatellite ms4760 haplotypes in the clinical samples from Kachin State, northeast Myanmar, 2007–2009*

Haplotype†	No. rep 1	No. rep 2	Haplotype frequency, %
1	2	2	12.7
3	1	2	5.1
5	4	1	3.8
6	2	1	13.9
7	3	1	54.4
9	3	2	3.8
14	3	1	1.3
18	2	2	2.5
34	4	1	1.3
35	1	1	1.3

*Pfnhe1, *Plasmodium falciparum* Na^+^/H^+^ exchanger. †Numbers refer to the haplotype list of Meng et al. (*5*).

## Conclusions

In Myanmar, high-level resistance to chloroquine and pyrimethamine–sulfadoxine was reported more than a decade ago (*11**–**13*). Our molecular survey showed that the major chloroquine resistance allele CVIET has reached fixation, and triple and quadruple mutations in *pfdhfr* and *pfdhps* were highly prevalent in this region. These findings strongly suggest that a large proportion of parasites might show clinical resistance to chloroquine and antifolate drugs. Although chloroquine has been withdrawn from treating *P. falciparum* malaria for decades in some regions, the *pfcrt* resistance alleles showed no sign of abating (*6*). Furthermore, despite adoption of artemisinin combination therapy in 2002, the frequency of highly mutated *pfdhfr* and *pfdhps* haplotypes appeared to have increased during this study, which suggested that artemisinin combination therapy might not have retarded the spread of antifolate-resistant parasites. This situation differs from that in the western Myanmar border area but is similar to that in Thailand and Cambodia, where highly mutated *pfdhfr* and *pfdhps* genotypes also were common (*14**,**15*).

Mutations in *pfmdr1* are associated with resistance to several antimalarial drugs including chloroquine, mefloquine, and quinine and increased *pfmdr1* copy number is responsible for mefloquine resistance. We found that ≈60% of the parasites contained the wild-type *pfmdr1* allele, similar to some parasites from the western Myanmar border area (*15*). No *pfmdr1* amplification was detected, suggesting that parasites from this region might be mefloquine sensitive, consistent with the fact that mefloquine has not been deployed here. In contrast, in vitro mefloquine resistance was observed in southeast Myanmar bordering Thailand (*13*), possibly because of the extensive use of mefloquine in Thailand for the past 2 decades.

Although the validity of *pfnhe1* minisatellite polymorphism for predicting quinine resistance remains uncertain and may depend on the parasites’ origins (*7**–**9*), we detected significant association of decreased quinine susceptibility with increased DNNND repeat copies (*5*). We have provided further evidence on the high prevalence of parasites with increased DNNND repeats in *pfnhe1*, which suggests that some parasite strains might show reduced sensitivity to quinine.

Overall, our molecular survey of antimalarial drug resistance in *P. falciparum* showed high frequency of multidrug-resistant haplotypes in northeastern Myanmar. Moreover, parasites in this region had unique multilocus genotypes that differed markedly from those in other areas of the GMS. These findings suggest that coordinated efforts are necessary to thwart the spread of resistant strains across larger geographic regions. Our molecular study showed only the genotypes of the drug resistance genes; further in vitro and in vivo studies are required to corroborate these findings.

## Supplementary Material

Technical Appendix Table showing primers used to amplify drug resistance-related genes in Plasmodium falciparum isolates.
