# Supplementary material for: Multidrug-Resistant Genotypes of Plasmodium falciparum, Myanmar
Source: Emerg Infect Dis. 2011 Mar;17(3):498–501. doi: 10.3201/eid1703.100870 (PMC3166001; doi:10.3201/eid1703.100870)
Supplement: Technical Appendix — Table showing primers used to amplify drug resistance-related genes in Plasmodium falciparum isolates. [file 10-0870-Techapp.pdf]

Article DOI: 10.3201/eid1703.100870

Multidrug-Resistant Genotypes of *Plasmodium falciparum*, Myanmar

Technical Appendix

Table. Primers used to amplify drug resistance–related genes in *Plasmodium falciparum* isolates, northeastern Myanmar, 2007–2009*

Target gene	Primer name	Sequences (5′ → 3′)	SNPs	Size of PCR product, bp	Reference
Pfcrt	Outer-PFCF	CCGTTAATAATAAATACAGGC		1,600	(*1*)
	Outer-PFCR	CTTTTAAAAATGGAAGGGTGT			
	Inner-pf72	TGTGCTCATGTGTTTAAACTTAT	72–76	552	
	Inner-pr72	AAAATAGTATACTTACCTATATCT			
	Inner-pf220	CTTATACAATTATCTCGGAGCAG	220	178	
	Inner-pr220	ATAATAAAAACAAAGTTTAAGTGT			
Pfmdr1	19F	AGAGAAAAAAGATGGTAACCTCAG	86, 184	590	(*2*)
	606R	ACCACAAACATAAATTAACGG			
	2902F	GCGGAGTTTTTGCATTTAGTTCAGATGATG	1034–1246	960	
	3867R	AGCAGCAAACTTACTAACACGTTTAACATC			
Pfdhfr	Outer F	TTTATATTTTCTCCTTTTTAT	16, 51, 59, 108, 164	665	(*3*)
	Outer R	TTACTAGTATATACA TCGCTAACAG			
	Inner F	ATGATGGAACAAGTCTGCGAC		646	
	Inner R	TTACTAGTATATACATCGCTAACA			
Pfdhps	Outer F	GGTATTTTTGTTGACCTAAACG		727	
	Outer R	TCCAATTGTGTGATTTGCCAC			
	Inner F1	GGTATTTTTGTTGAACCTAAACG	436, 437	319	
	Inner R1	CTGGATTATTTGTACAAGCAC			
	Inner F2	GAATGTGTTGATAATGATTTAG	540, 581, 613	472	
	Inner R2	TCCAATTGTGTGATTTGTCCAC			
Pfnhe1	3802F	TTATTAAATGAATATAAAGA	Ms4760	520	(*4*)
	4322R	TTTTTTATCATTACTAAAGA			

*SNP, single nucleotide polymorphisms; *Pfcrt*, *Plasmodium falciparum* chloroquine resistacne transporter; *Pfmdr1*, *P. falciparum* multidrug resistance 1; *Pfdhfr, P. falciparum* dihydrofolate reductase; *Pfdhps, P. falciparum* dihydropoteroate synthase; *Pfnhe1*, *P. falciparum* Na^+/H^+ exchanger.

References

1. Mittra P, Vinayak S, Chandawat H, Das MK, Singh N, Biswas S, et al. Progressive increase in point mutations associated with chloroquine resistance in *Plasmodium falciparum* isolates from India. J Infect Dis. 2006;193:1304–12. PubMed DOI: 10.1086/502979

2. Basco LK, Ringwald P. Molecular epidemiology of malaria in Cameroon. X. Evaluation of PFMDR1 mutations as genetic markers for resistance to amino alcohols and artemisinin derivatives. Am J Trop Med Hyg. 2002;66:667–71. PubMed

3. Gebru-Woldearegai T, Hailu A, Grobusch MP, Kun JF. Molecular surveillance of mutations in dihydrofolate reductase and dihydropteroate synthase genes of *Plasmodium falciparum* in Ethiopia. Am J Trop Med Hyg. 2005;73:1131–4. PubMed

4. Ferdig MT, Cooper RA, Mu J, Deng B, Joy DA, Su XZ, et al. Dissecting the loci of low-level quinine resistance in malaria parasites. Mol Microbiol. 2004;52:985–97. PubMed DOI: 10.1111/j.1365-2958.2004.04035.x
